# The Treatment of Fibrosis of Joint Synovium and Frozen Shoulder by Smad4 Gene Silencing in Rats

**DOI:** 10.1371/journal.pone.0158093

**Published:** 2016-06-28

**Authors:** MingFeng Xue, SuiLiang Gong, JiaPing Dai, Gang Chen, JunYu Hu

**Affiliations:** Department of Orthopaedic Surgery, Jiaxing Second Hospital, Jia Xing, 31400, China; University of Alabama at Birmingham, UNITED STATES

## Abstract

Soft tissue fibrosis at the joint induced by inflammation is the pathological basis of frozen shoulder. In the present study, we utilized a lentiviral approach to silence the Smad4 gene in an in vitro fibrosis model of fibroblasts and an in vivo frozen shoulder model. We observed the change in the fibrosis process and the biological indicators of frozen shoulder. The in vitro fibrosis models (Rat myoblasts L6, Rat synovial cell RSC-364 and Rat chondrocytes RCs) were established using TGF-β1 induction, and the effect of Smad4 gene silencing on fibrosis was analyzed. The method of Kanno A was employed to establish a rat model of frozen shoulder, and Smad4 in the relevant part was knocked down with the lentiviral approach. We then examined the abduction and rotation angles and the length of synovial intima and measured the inflammatory factors in effusion and the fibrotic markers of tissues. We found that Smad4 knockdown suppressed the proliferation and expression of fibrotic markers in L6, RSC-364 and RCs cells induced by TGF-β1. MMP activity measurements showed that Smad4 knockdown significantly reversed the decrease in MMP activity in these three cell lines that were induced by TGF-β1. Furthermore, using lentivirus in the rat frozen shoulder model, we found that Smad4 silencing attenuated the inflammatory response and fibrosis. It significantly inhibited the increase of the Vimentin, α-SMA, collagen I and III, Lama1 and Timp1 proteins in synovial tissue as well as the inflammatory factors of TNF-a, IL-1α/β, IL-6 and IL-10 in effusion. MMP acidity assays revealed that Smad4 silencing inhibited MMP activity in the synovial, cartilage and ligament tissues in the model animals. The assessment of the phosphorylated Smad2/3 in the nuclei isolated from the synovial tissues showed that Smad4 silencing significantly inhibited the phosphorylation and subsequent nuclear translocation of Smad2/3 proteins. Moreover, Smad4-shRNA lentivirus inhibited the decrease in both the abduction and rotation angles caused by immobilization as well as the decrease in the length of the synovial intima. Based on shoulder movement data, Smad4 knockdown can increase the rotation limitation caused by immobilization. In summary, Smad4 silencing can suppress chronic inflammation and fibrosis in joint tissues by inhibiting the TGF-β/Smad pathway and can play a positive role in the prevention and treatment of joint stiffness.

## Introduction

Frozen shoulder, also called scapulohumeral periarthritis, is a progressive condition in the shoulder joint characterized by pain and a limited range of movement. Though the cause of a primary frozen shoulder remains unclear, it typically has three stages over a period of approximately 12–40 months (average, 30 months: The painful stage, the frozen stage and the thawing stage. Some patients recover joint motion after that time period, but a considerable number of patients have apparent residual limitations in shoulder motion that impact their daily work and life. The most distinctive clinical feature of frozen shoulder is the limitation of active and passive movement of the shoulder joint in all directions, particularly external rotation.

Transforming growth factor-β (TGF-β) is a multifunctional cytokine that plays a definitive role in the expression of extracellular matrix proteins; the proliferation, differentiation, and apoptosis of cells; and immunity. TGF-β has been proven to contribute to fibrosis[1], and it can promote the formation of an extracellular matrix; the synthesis of collagen, fibronectin and protein polysaccharide by fibroblasts; and the expression of specific surface membrane receptors for extracellular matrix proteins, as well as the transformation from epithelial cells to mesenchymal cells. Studies have shown that TGF-β plays a crucial role in the development of such diseases as hepatic fibrosis[2] and pulmonary interstitial fibrosis[3]. In addition, it has been found that the expression of TGF-β and its receptor is increased in the shoulder joint capsule of patients suffering from primary or secondary frozen shoulder, indicating the important role of joint capsule fibrosis in the genesis and development of frozen shoulder[4]. As receptor kinases downstream of TGF-β, Smad protein family members play indispensable mediatory roles in the TGF-β signal pathway. To date, eight Smad family members have been found in mammals. In terms of their structures and functions, they are divided into three groups. Smad4, the common partner for all R-Smads, interacts with R-Smads to form stable heteromultimers, which are translocated into the nucleus and regulate transcription of target genes. As a pivotal protein in the signal pathway, Smad4 interacts with other Smad proteins and serves as the key link in the signal transduction pathway of the TGF-β superfamily to regulate downstream effectors or crosstalk pathways, thereby playing a pivotal role in signaling process.

Many researchers have found that the TGF-β/Smad signal pathway is closely related to fibrosis in various tissues. Hao J et. al found that TGF-β, Smad2/3 and Smad4 increased in the scar tissue after myocardial infarction[5]. Kopp J et. al found that increased Smad7 could inhibit the expression of collagen I and α-SMA and impair scar hyperplasia[6]. Goto Y found that an increase in Smad4 promotes fibrogenesis in mouse glomeruli[7]. Fibrosis of the joint capsule is a major pathological change in frozen shoulder, and synovial fibrosis is one of its main characteristics. In addition, dysfunction of osteocytes is also a marker event of frozen shoulder. In the present study, we investigated whether Smad4 gene silencing could inhibit fibrosis of synovial tissue regulated by the TGF-β/Smad pathway and produce a positive effect on frozen shoulder. As a critical protein, Smad4 mediates many pathways. The BMP/Smad4 pathway, for example, is closely associated with frozen shoulder. BMPs are multifunctional glycoproteins of the TGF-β family, among which BMP-2 plays a role in ectopic bone formation, the regulation of cell differentiation and migration, and the proliferation and apoptosis of fibroblasts and hyperplasia of joint synovia. Therefore, the activation of both the TGF-β/Smad and BMP-2/Smad pathways plays a positive regulatory role in the pathological injury of frozen shoulder.

We employed TGF-β1-induced myoblasts, synovioblasts and rat chondrocytes to establish in vitro fibrosis models and used the Kanno A method to establish a rat model of frozen shoulder. Based on these models, we knocked down Smad4 with a viral approach and investigated the effect of Smad4 knockdown on fibrosis and frozen shoulder. The results show that Smad4 plays an important and crucial role in the pathological development of fibrosis and frozen shoulder. Specific silencing of Smad4 can inhibit fibrosis regulated by the TGF-β/Smad signal pathway and block the activation of the TGF-β/Smad pathway, which may impair the genesis and development of frozen shoulder. Smad4 knockdown significantly reduced the translocation of phosphorylated Smad2/3 and, subsequently, suppressed the transcription of genes downstream of Smad2/3. According to our results, Timp1, a gene regulated by Smad2/3, was inhibited by Smad4 silencing, which in turn increased the activity of MMPs. This may be one of the mechanisms through which Smad4 silencing inhibits fibrosis. Moreover, the signal pathway responsible for the proinflammatory effect of TGF-β was also interrupted by Smad4 knockdown.

## Materials and Methods

### Cell culture

Rat myoblasts L6 and rat synovial cells RSC-364 were purchased from the cell bank of the Chinese Academy of Sciences; RC cells (primary chondrocytes at passage 3) were purchased from Shine Star (Hubei) Biological Engineering Co., Ltd.; and the viral packaging cell line 293 TN, was purchased from Systermbio. All cells were maintained in DMEM (Dulbecco minimum essential medium, Invitrogen, CA, USA) containing 10% FBS (fetal bovine serum, Invitrogen, CA, USA) at 37°C in a humidified 95% air and 5% CO_2_ incubator (Sanyo, Japan). All cells grew adhesively and were passaged using 0.25% trypsin digestion when the coverage reached 80%. All cells first received at the laboratory were marked as P1 and only cells at a passage less than three were utilized in experiments.

### Construction of pLV-shRNA-Smad4 plasmids

A shRNA sequence complementarily binding to rat Smad4 mRNA (NM_019275.2) was selected. The target sequence of shRNA (5’- GCTGTCCTATTGTAACTGT-3’) is homologous to nt 1031–1049 of Smad4 mRNA. The oligonucleotide templates of this shRNA were chemically synthesized and cloned into the linear pSIH1-H1-copGFP shRNA vector (System Biosciences, CA, USA), which was obtained through digestion by BamH I and EcoR I and purified by agarose gel electrophoresis. A scrambled sequence (5’-GAAGCCAGATCCAGCTTCC-3’) was used as a negative control (NC). Sequencing was used to confirm the vectors that were constructed (pSIH1-shRNA-Smad4 and pSIH1-NC).

### Lentivirus packaging

One day before transfection, 293TN cells were seeded into 10-cm dishes. In accordance with the manufacturer’s protocol, 2 μg of each pSIH1-shRNA vector or pSIH1-NC and 10 μg pPACK Packaging Plasmid Mix (System Biosciences) were co-transfected using Lipofectamine2000 (Invitrogen). The medium was replaced with DMEM plus 1% FBS. Forty-eight hours later, the supernatant was harvested, cleared by centrifugation at 5000×g at 4°C for 5 minutes, and passed through a 0.45 μm PVDF membrane (Millipore, MI, USA). The titer of the virus was determined by gradient dilution. The packaged lentiviruses were named Lv-shRNA-Smad4 and Lv-NC.

### Establishment of in vitro cellular model of fibrosis by TGF-β1 induction

L6 cells, RSC-364 cells and RCs in the logarithmic growth phase were prepared into cell suspensions and subjected to viable cell counting with Trypan blue. The cells were re-suspended in DMEM plus 10% FBS to a concentration of 1×10^5^/mL and seeded to six-well plates with 2 mL per well. The cells were cultured at 37°C and 5% CO_2_ for 24 hours. The medium was refreshed, and the model groups were added with recombinant TGF-β1 protein (Abcam, USA) at a final concentration of 10 ng/mL. After being cultured for 72 hours under normal conditions, the cells were collected and subjected to measurements of Smad4 mRNA and protein, as well as fibrosis-relating proteins, Vimentin, α-SMA and collagen I, to verify whether the model was successfully established. At the same time, cell proliferation was detected at different time points with CCK-8 (cell counting kit-8, Dojindo, Japan) assays.

### Detecting inhibition of fibrosis by Smad4 silencing in vitro

We employed the lentivirus to knock down Smad4 in the three cell lines. On the day before infection, 1×10^5^ cells in 2 mL suspension were seeded to each well of 6-well plates. The cells were divided into the following groups: (1) Cells (blank cell); (2) Cells +Lv-NC (cells infected with the lentivirus expressing scrambled sequence); (3) Cells +Lv-shRNA (cells infected with the lentivirus expressing shRNA targeting Smad4). One day after seeding, the cells were infected with lentivirus diluted by DMEM supplemented with 10% FBS at a multiplicity of infection (MOI) of 10, and the medium was refreshed 24 hours later. The infection was confirmed by fluorescence microscopy detecting GFP (Green Fluorescent Protein) 72 hours after infection. A quantitative PCR analysis was employed to detect the mRNA level of Smad4. We treated the control cells and the Smad4-silenced cells with TGF-β1 at a final concentration of 10 ng/mL, harvested the cells 72 hours later, and extracted the total protein to measure Vimentin, α-SMA and collagen I. The proliferation changes were monitored using CCK-8. To elucidate the mechanism and pathway through which Smad4 silencing affects cellular fibrosis, we examined the expression level of Timp1, a key protein mediating cellular fibrosis, and MMP activity.

### Detection of cell viability by CCK-8 assay

Cell viability was detected with a CCK-8 assay after 24, 48 and 72 hours as follows: 10 μL CCK-8 solution was added into each well, cells were incubated at 37°C, and 5% CO_2_ for 4 hours, and the absorbance value was detected at 450 nm with a plate reader (Thermo, USA).

### Establishment of rat frozen shoulder model and in vivo gene intervention

Sprague-Dawley rats, male, 12 weeks old, were used in the study. The animals were purchased from SLRC Laboratory Animal Co., Ltd and maintained in the animal experiment center of the Second Military Medical University. The Second Military Medical University’s Animal Care and Use Committee approved this study. In accordance with the method of Kanno A et. al[8], the shoulder model of rats was established by immobilizing their glenohumeral joints. Briefly, under anesthesia, a longitudinal skin incision was made parallel to the humeral shaft, of which the lateral aspect was exposed, and from which the insertion of the deltoid muscle was separated. Perpendicular to the scapular spine, another incision was made and both infraspinatus and subscapularis muscles were slightly lifted from the scapular body. The shoulder was then fixed by using two plastic plates with prethreaded soft flexible wires, one for the ventral side and the other for the dorsal side, and the two flexible wires, passing through a bony hole, were twisted and tied to fasten the scapular body with the plates which were fixed using two screws. The shoulder joint was secured at 60° abducted position. Postoperatively, the rats were allowed unlimited movement and free access to water and food. Six weeks after immobilization, the range of passive motion and synovium length change were measured in the glenohumeral joints on three rats from the model group or the sham group to determine whether the model was established. After the model was established, all implants being removed after euthanasia, the animals then received drug administration. The rats were divided into four groups: sham group (12 rats), model + saline group (12 rats), model + Lv-NC group (12 rats), and model+ Lv-shRNA-Smad4 group (12 rats). The rats in the sham group also received the operation except that only screws were inserted into the humerus. Administration was performed by injecting 50 μL saline (sham group and model+saline group) or viral solution (5×10^7^ifu) (model+Lv-NC group and model+Lv-shRNA-Smad4 group) in the shoulder joint capsule once every three days. During the last 15 days in month one, two or three after medication, joint effusion was collected from four rats of each group, before each injection, stored and pooled (five collections from each rat, with a total of 50–100 μL) for ELSIA measurements of TNFa, IL-1a/β, IL-6 and IL-10. After the effusion collection, the rats were sacrificed by cervical dislocation, and the range of passive motion and synovium length change were measured in the glenohumeral joints. The joint synovial tissues were collected. One portion was frozen for GFP expression detection under a fluorescence microscope, which was used to determine the infection efficiency of tissues. Another portion was used for protein extraction and western blotting of TGF-β, α-SMA, collagen I and III, lama 1 and Timp1 and for the examination of Smad2/3 phosphorylation and nuclear translocation. In addition, joint cartilage and ligament tissues were collected and subjected to protein extraction and an MMP activity assay. Both shoulder girdles were collected and stored at -80°C until testing. In each group, abduction and rotation angles were measured on 6 rats, and the other 6 rats were used for measurement of the length of synovial intima.

### mRNA quantification

Total RNA was isolated with Trizol Reagent (Invitrogen) according to the manufacturer’s instruction and reversely transcribed into cDNA using M-MLV Reverse Transcriptase (Takara BIO, Japan) and oligo (dT)18 primer (Takara BIO). The following specific primers were used in the quantitative PCR of rat Smad4 and β-actin: Smad4: 5’- TGGACATTACTGGCCGGTTCACAA-3’ and 5’-GCCAAGCAAAAGCGATCTCCTCCAG -3’ and β-actin: 5’-CCTGTACGCCAACACAGTGC-3’ and 5’-ATACTCCTGCTTGCTGATCC-3’. The lengths of the amplified products were 219 bp and 211 bp, respectively. Real-time PCR was performed using a SYBR® Premix Ex Taq™ kit (Takara BIO) and a TP800 System (Takara BIO). cDNA from 200 ng total RNA was used as the template. The PCR reactions was carried out under the following conditions: 40 cycles of denaturation at 95°C for 10 s, annealing at 60°C for 20 s and extension at 72°C for 20 s. The mRNA levels of Smad4 were normalized using the delta-delta Ct method to the expression of β-actin, an endogenous housekeeping gene.

### Detection of protein content in cells or tissues

The total protein was extracted from the cells using the M-PER mammalian protein extraction reagent (Pierce, IL, USA) or from rat joint synovial tissues using the T-PER tissue protein extraction reagent (Pierce). Equal amounts of protein (25 μg per lane), estimated by a bicinchoninic acid (BCA) protein assay kit (Pierce), were loaded onto (11%) SDS-PAGE gels and transferred onto nitrocellulose membranes. The blots were probed with a monoclonal antibody against rat Smad4 (1:300), TGF-β (1:800), α-SMA (1:600), collagen I (1:800), collagen III (1:600), Lama1 (1:800), Timp1 (1:800) and beta actin (1:1200) (Santa Cruz, USA), followed by the secondary HRP-conjugated anti-mouse/rabbit antibody (Santa Cruz, USA). After washing, the bands were detected by chemiluminescence and imaged with X-ray films. Beta actin was used as an endogenous reference for normalization.

### Measurement of phosphorylated Smad2/3 in cell nuclei

Nuclear proteins were extracted from the frozen rat joint synovium samples using the CelLytic™ NuCLEAR™ Extraction Kit (Sigma, USA), followed by western blotting of phosphorylated Smad2/3 (for primary antibodies, the dilution ratio of antibody against P-Smad2 was 1:750; for P-Smad3, it was 1:1000) (CST, USA). The detection and other experimental conditions were the same as those used for other proteins.

### Measurement of abduction and rotation angles and length of synovial intima

Measurements were carried out as previously described[8]. Briefly, the scapula was fixed on the measuring device with the center of its humeral head fixed to the center of the rotation of the device. To measure the abduction and rotation angles, a torque of 10 gram-weight was applied to the humeral shaft and the forearm, respectively. Radiographs were taken to measure the abduction and rotation angles using Image J 1.37 software. The angle between lines 1 (the line from the medial edge of the scapular spine to the midpoint of the segment that binds the upper and lower glenoid edge) and 2 (the line from the center of the humeral head to that of the humeral condyle) was defined as the abduction angle and the angle between lines 3 (the line from the medial edge of the scapular spine to the midpoint of the segment that binds the anterior and posterior glenoid edge) and 4 (the line from the midpoint of the segment that binds the anterior and posterior edge of the wrist joint to the most proximal site of the ulna) was defined as the rotation angle. For quantification of the extent the intra-articular adhesion in a histologic assessment, we also measured the length of the synovial intima using Image J. The attachment site of the synovial intima on the humeral neck to that on the inferior edge of the glenoid was used for length.

### Measurement of inflammatory factors in joint effusion by ELISA

The effusion samples were removed from -80°C and incubated at 4°C for 10 min to thaw. ELISA kits (Invitrogen, USA) were used to measure the TNFa, IL-1a/β, IL-6 and IL-10 levels in the samples, following the manufacturer’s instructions. The differences among the sham group, model + saline group, model + Lv-NC group and model+ Lv-shRNA-Smad4 group were analyzed to evaluate both the relationship between frozen shoulder and inflammatory conditions and the effect of Smad4 depletion on possible inflammation.

### Measurement of activity of MMPs

The cells were treated with TGF-β1 for 72 hours, and the rat synovium, cartilage and ligament were lysed with RIPA. The total proteins were quantified with the BCA assay, and MMP activity was measured with the MMP Activity Assay (Fluorometric-Red) (Abcam, USA).

### Statistical analyses

All data are expressed as mean±SD, and analyzed by one way ANOVA. Least Significant Difference (LSD) was used for multiple comparisons between any two means. P-values <0.05 were considered statistically significant. All statistical analysis was performed using SPSS 13.0 software.

## Results

### Lentiviral mediated RNA interference in L6, RSC-364 and RC cells

**Fig 1:**
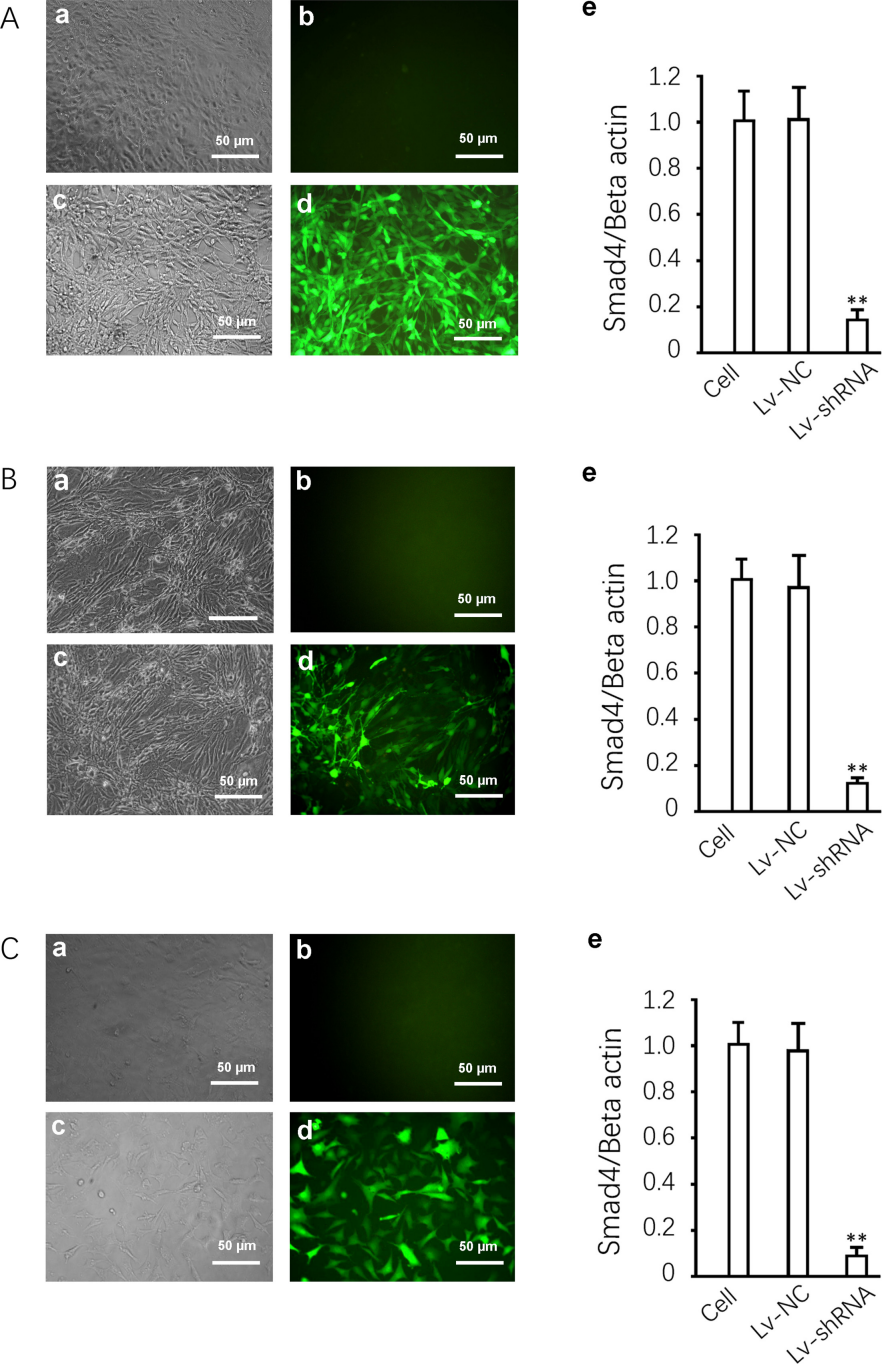
Lentiviral mediates Smad4 knockdown in L6, RSC-364 and RC cells. (A) Left, representative photo of GFP expression in L6 infected with Lv-shRNA-Smad4 and the uninfected L6 cells for gene delivery efficiency assessment 72 hours after infection. Right, results of Smad4 mRNA quantification. (B) Left, representative photo of GFP expression in RSC-364 infected with Lv-shRNA-Smad4 and the uninfected RSC-364 cells for gene delivery efficiency assessment 72 hours after infection. Right, results of Smad4 mRNA quantification. (C) Left, representative photo of GFP expression in RC infected with Lv-shRNA-Smad4 and the uninfected RC cells for gene delivery efficiency assessment 72 hours after infection. Right, results of Smad4 mRNA quantification. ** *P* < 0.01 compared to the Lv-NC group.

Recombinant lentiviruses were constructed successfully and were used for the infection of L6 (Fig 1A), RSC-364 (Fig 1B) and RC cells (Fig 1C) at a MOI of 10, producing a high transfection efficiency. GFP expression was observed by fluorescence microscopy (Fig 1A, 1B, 1C, 1Cb and 1Cd), and the comparison between the cells expressing fluorescent protein and the total number (Fig 1A, 1B, 1C, 1Ca and 1Cc) showed a delivery efficiency of close to 100%. There was no GFP expression observed in the control group (Fig 1A, 1B, 1C, 1Ca and 1Cb). Real-time PCR results showed that shRNA significantly decreased Smad4 mRNA in the three cell lines compared with the uninfected group (*P* < 0.01, Fig 1A, 1B, 1C and 1Ce). Meanwhile, the Lv-NC had no significant effect on Smad4 mRNA, showing that the viral system itself did not alter the expression of the target gene. For mRNA level assays, three independent experiments were performed on three parallel samples, each sample run in three tubes for PCR.

### Effects of Smad4 gene silencing on fibrosis in L6, RSC-364 and RC cells induced by TGF-β1

**Fig 2:**
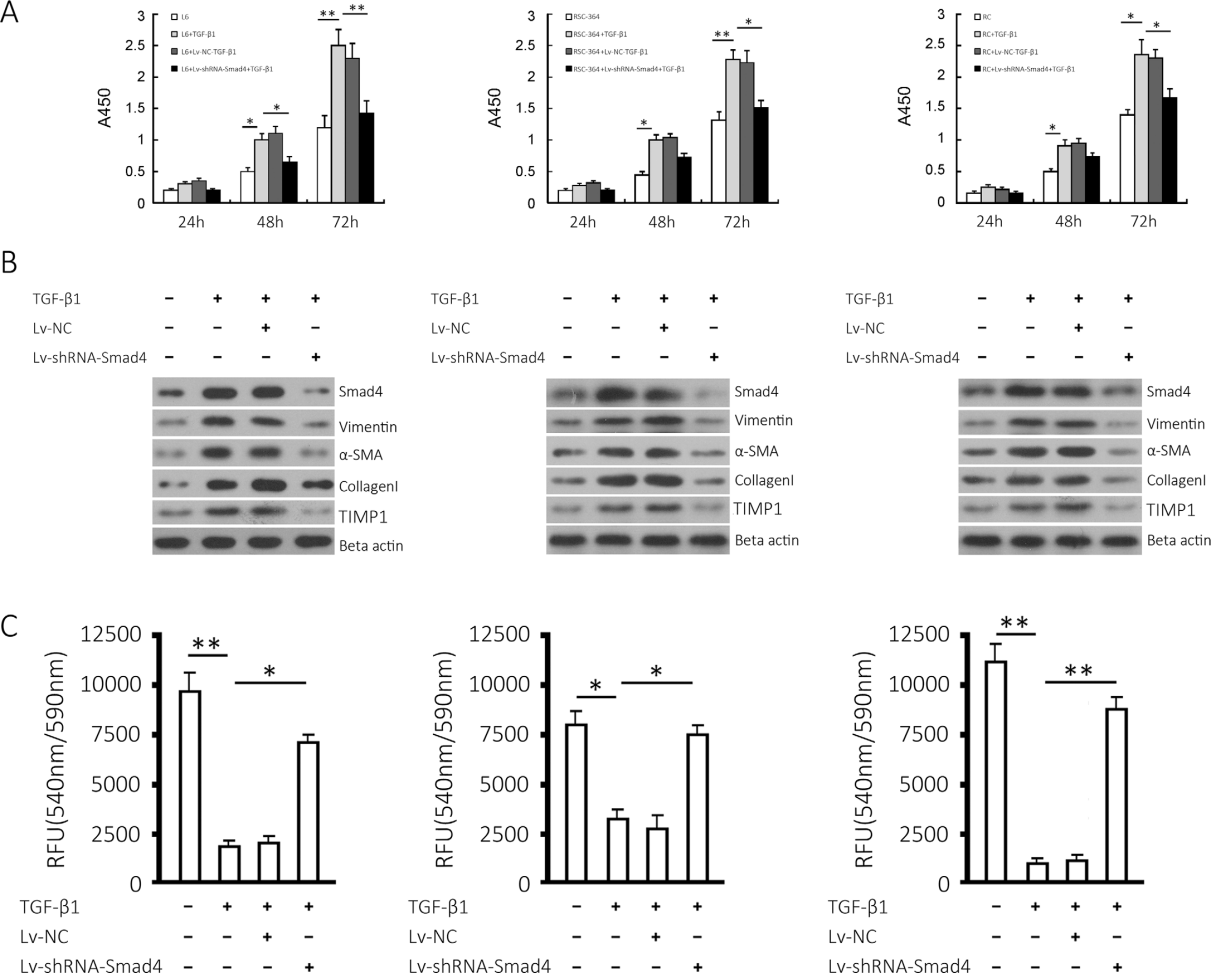
Effects of Smad4 knockdown on TGF-β1-induced proliferation and fibrotic markers in L6, RSC-364 and RC cells. (A) Cell proliferation of the three cell lines and respective Smad4-silenced cell lines cultured in the presence or absence of TGF-β1 at the indicated time points. (B) Smad4, Vimentin, α-SMA, collagen I and Timp1 proteins expressed in L6, RSC-364 and RC cells and the respective Smad4-silenced cells induced by TGF-β1 were detected. (C) MMP activity was measured in the three cell lines and respective Smad4-silenced cell lines. Left, middle, right show the results from L6, RSC-364 and RC cells, respectively. The results are shown as the mean ± SD of at least 3 separate experiments. * *P* < 0.05 and ** *P* < 0.01 compared to the indicated group.

L6, RSC-364 and RC cells were infected with recombinant lentiviruses; re-seeded after 48 hours; and stimulated with 10 ng/mL TGF-β1 for 24, 48 or 72 hours. Cell viability was assessed using CCK-8. The results (Fig 2A) showed that 10 ng/mL TGF-β1 increased the proliferation of L6, RSC-364 and RC cells at the log phase. There was a significant difference between the viability of the TGF-β1-induced group and the control group (*P* < 0.05). Smad4 silencing attenuated the increase in proliferation induced by TGF-β1 for 48 or 72 hours (*P* < 0.05), but there was no observable change in the proliferative activity of Lv-NC + TGF-β1 (*P* < 0.05, compared with TGF-β1 Group). These results suggest that silencing Smad4 significantly inhibits the increased proliferation of the three groups of cells induced by TGF-β1. Western blotting results (Fig 2B) showed that the stimulation of 10 ng/mL TGF-β1 increased Smad4, Vimentin, α-SMA, collagen I and Timp1 in the three groups of cells, and Smad4 knockdown impaired the increase in these three proteins. As shown in Fig 2C, TGF-β1 treatment (10 ng/mL, 72 hours) significantly decreased the activity of MMPs in L6, RSC-364 and RC cells (*P* < 0.05), which was reversed by Lv-shRNA-Smad4 (*P* < 0.05) but not Lv-NC infection, suggesting that Smad4 gene knockdown offset the decrease of MMP activity in the three cell lines induced by TGF-β1. Three independent experiments were performed in triplicate.

### Infection efficiency in tissue and effects of Smad4 silencing of relevant proteins

**Fig 3:**
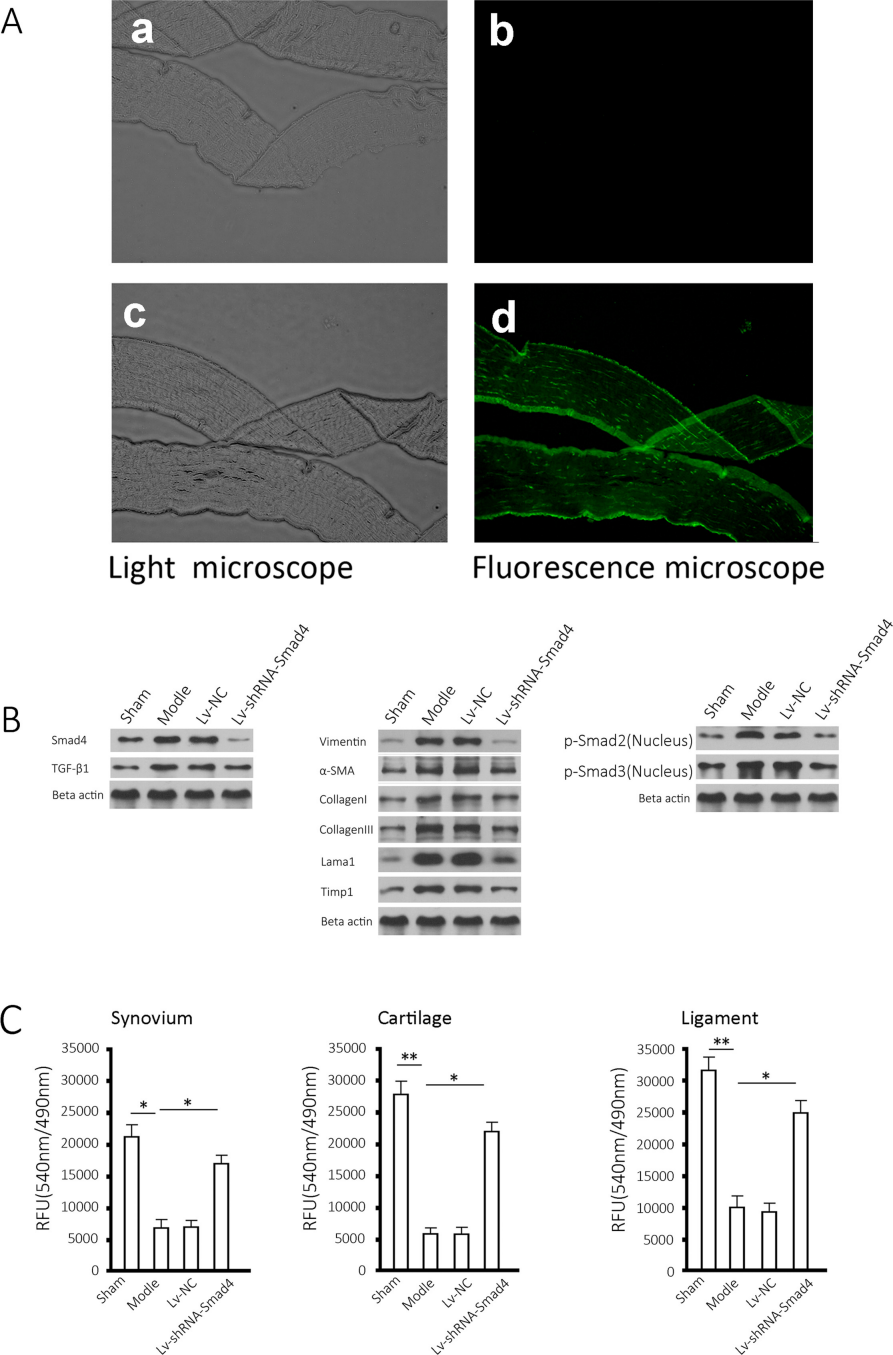
Effects of Smad4 expression lentivirus on fibrotic markers. (A) A typical section was observed in visible light and with fluorescence microscopy, respectively (upper, synovial tissue from control group; lower, synovial tissue from the group of Lv-shRNA-Smad4, all samples collected at month 3). (B) Protein extracted from rat joint synovial tissues was detected for Smad4, TGF-β, Vimentin, α-SMA, collagen I, collagen III, Lama1, Timp1 and Beta actin as well as nuclear translocation of Smad2/3, all samples collected at month 3. (C) Three types of tissues from the rats undergoing the indicated treatment for 3 months were separated and measured for MMP activity. The results are shown as the mean ± SD of at least 3 separate experiments. * *P* < 0.05 and ** *P* < 0.01 compared to the indicated group.

The gene delivery efficiency was evaluated through observation of GFP in frozen sections of synovial tissues of rat joints (Fig 3A). As shown in the figure, the injection of lentivirus into the joint capsule effectively delivered genes to the tissues (Fig 3A, 3Ac and 3Ad), but no fluorescent signal was detected in the tissue of the control group (Fig 3A, 3Aa and 3Ab). Western blotting results showed that compared with the sham group, Smad4 and TGF-β1 increased in the model group’s joint synovial tissues (Fig 3B, left), and the fibrosis markers, including Vimentin, α-SMA, collagen I and III, Lama1, and Timp1 increased in a similar way (Fig 3B, middle). In the model group, phosphorylated Smad2/3 accumulated in the cellular nuclei (Fig 3B, right), and MMP activity in the joint synovium decreased significantly compared with the control group (*P* < 0.05). As expected, Smad4 silencing increased MMP activity (*P* < 0.05) in synovial tissue (Fig 3C, left), as well as in cartilage and ligaments (Fig 3C, middle and right).

### Measurements of TNFa, IL-1a/β,IL-6 and IL-10

**Fig 4:**
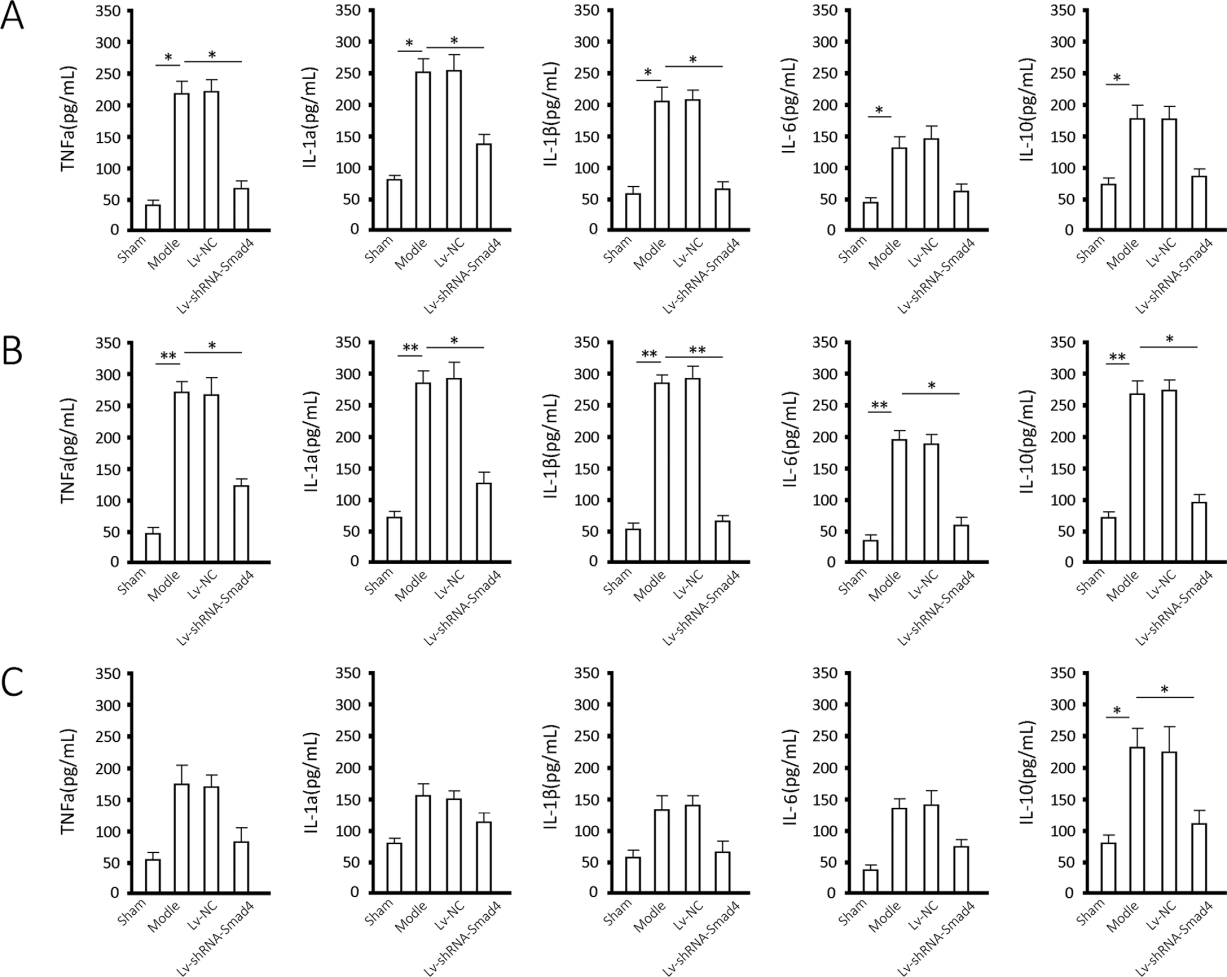
TNFa, IL-1a/β, IL-6 and IL-10 level in Effusion. Samples were extracted from the joints of rats undergoing the indicated treatment at the end of each month for 3 months (Fig 4A, month 1; Fig 4B, month 2; Fig 4C, month 3) and were measured for TNFa, IL-1a/β, IL-6 and IL-10 using ELISA. The results are shown as the mean ± SD for at least 3 separate experiments. * *P* < 0.05 and ** *P* < 0.01 when compared to the indicated group.

The measurements of TNFa, IL-1a/β, IL-6 and IL-10 in joint effusion (Fig 4) showed that there was a significant inflammation response in the frozen shoulder model, and the five cytokines increased significantly at the end of the first month after the model construction (*P* < 0.05). These inflammatory factors further increased significantly at the end of the second month (*P* < 0.05) and decreased during the following month, at the end of which only IL-10 and IL-1a and IL-6 had significantly increased (*P* < 0.05). The depletion of Smad4 significantly decreased these five inflammatory factors by the end of the second month (*P* < 0.05) and decreased IL-10 at the end of the third month (*P* < 0.05) in the model group. The animal experiments were performed with four animals as biological replicates, and two parallel wells were used in ELISA assay of each sample.

### Measurement of the range of motion and the length of synovial intima

**Fig 5:**
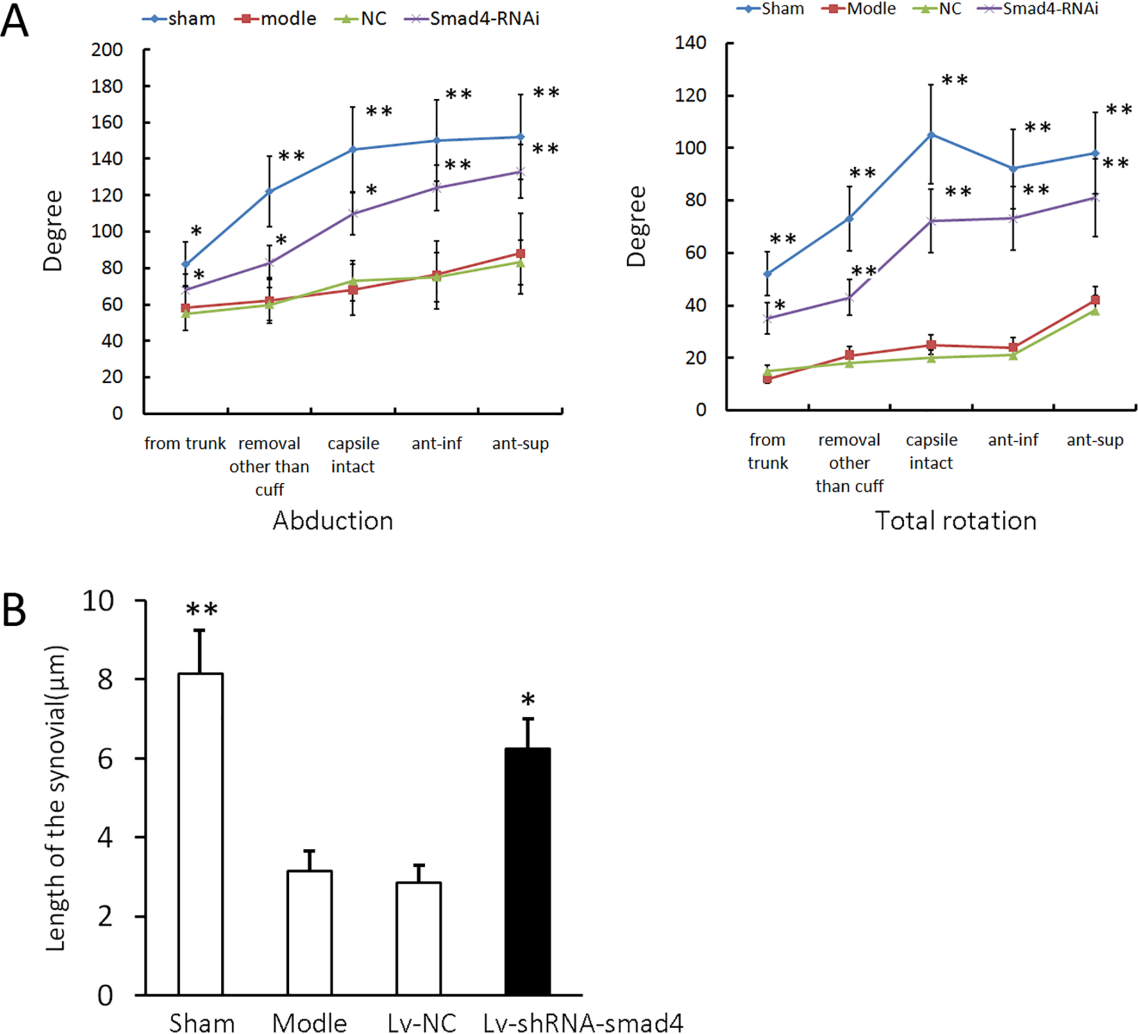
Smad4 shRNA improved the decreased abduction, total rotation, and decreased length of synovial intima by immobilization. (A) The abduction and total rotation detected in the sham, model, NC, and Smad4 shRNA groups, samples from the groups undergoing three months of treatment. From trunk, the shoulder is removed from the trunk; removal other than cuff, after removal of the muscles other than the rotator cuff muscles; capsule intact, removal of the rotator cuff muscles with the joint capsule intact; ant-inf, after anteroinferior capsulotomy; and ant-sup, after anterosuperior capsulotomy. (B) The lengths of synovial intima in the sham, model, NC and Smad4 shRNA groups. The error bars represent the standard deviation. *P < 0.05 and ** *P* < 0.01 compared with the immobilized group.

The range of passive motion is shown in Fig 5A. Compared to the sham group, the immobilized group showed a significant decrease in both the abduction and rotation angles, indicating that the model was successfully established. The administration of Smad4-shRNA lentivirus, but not NC lentivirus, attenuated the decrease caused by the immobilization in both abduction and rotation. Moreover, compared with the sham group, immobilization decreased the length of the synovial intima, and Smad4 knockdown partly reversed the decrease (*P* < 0.05), but the NC had no observable effect (Fig 5B). The animal experiments were performed with six animals as biological replicates, and the results are the mean of five measurements of each sample.

## Discussion

The definitive cause of frozen shoulder remains unclear, and one-fifth of patients have frozen shoulder in the opposite shoulder. Understanding the pathological changes in the joint capsule during the progress of frozen shoulder has important implications for treatment. Currently, the majority of researchers think that the major pathological changes in frozen shoulder are contracture and incrassation of the shoulder joint capsule, which are caused by lesions in the coracohumeral ligament (CHL), an important structure in the rotator interval. It is controversial whether this is a process of inflammation or fibrosis. Some researchers think that frozen shoulder is an inflammatory response, based on the inflammatory factors detected in shoulder joint capsule[9]. By contrast, some researchers have only found fibrosis change, contracture and incrassation of tissues in the shoulder joint capsule but no inflammatory factor, and have concluded that frozen shoulder resulted from fibrosis of joint capsule. Studies carried out in China also showed that fibrosis dominates in the development of frozen shoulder[4]. The contracture of rotator interval tissues plays an important role in the pathogenesis of frozen shoulder. The CHL is strained during external rotation of the shoulder joint, and contracture of different degrees occurs in the CHL in frozen shoulder. CHL contracture is the major pathological change of frozen shoulder, which mainly consists of compact collagen III fibers that are rich in fibroblasts and myofibroblasts, and the surgical release of CHL contracture can relieve pain and improve the movement of the shoulder joint[10–11].

TGF-β is the most potent pro-fibrotic factor and plays a key role in a variety of fibrotic environments[12–15]. Smad4, a key protein in the signaling pathway, interacts with different Smad proteins and acts as a key link in the TGF-β superfamily signal transduction pathway. As a pivot in the entire pathway, Smad4 is extremely important in the TGF-β-Smad signal transduction pathway[16–18]. Blocking Smad4 gene expression in relevant tissues certainly affects the TGF-β/Smad signal pathway, which is closely related to joint capsule fibrosis. Whether this effect can affect related lesions and associated proteins in frozen shoulders is the major question to be investigated in the study. We used three cell lines that are closely related to joint stiffness for in vitro experiments: rat myoblasts L6, rat synovial cells RSC-364 and rat chondrocytes. In vitro experimental data show that Smad4 gene knockdown can decrease the TGF-β1-induced proliferation of L6, RSC-364 and RC cells and the expression of Vimentin, α-SMA, collagen I and Timp1. TGF-β1 induced a decrease in MMP activity in the cells, which was offset by Smad4 knockdown. Moreover, the change in MMP was negatively correlated with that of the Timp1 protein. In addition, the in vivo data suggest that the effects of Smad4 gene knockdown include the inhibition of both the inflammatory response and fibrosis caused by immobilization and the partial reversal of the decrease in the length of synovial intima. For treatment of this disease, which is of an unclear cause and has few treatment approaches, this is obviously encouraging. We focused our study on the fibrosis change of synovium because the injection of lentivirus in the joint cavity can produce potent gene intervention in synovial tissue relative to other tissues. In the in vivo study, we observed significant changes in synovial fibrosis indices, indicating a causal relationship between synovial fibrosis and frozen shoulder. After genetic interference and model establishment, we extracted approximately 50 μL joint effusion at the end of each month and assessed TNFa, IL-1a/β /IL-6 and IL-10. The results showed that in the model group, these inflammatory factors increased significantly in the first and second months, and Smad4 interference impaired this trend. In the third month, the trend was similar as in the previous two months, but the differences narrowed and only an inter-group difference existing in the IL-10 level. The possible reason may be that Smad4 depletion inhibited the sustained increasing inflammatory response: from the second month to the third month. With suppressed inflammation, the inflammatory factors were decreased to some extents, and the decrease of the anti-inflammatory factor, IL-10, was significantly lower than that of the pro-inflammatory factors, indicating a drop of inflammatory response. The significant difference of the IL-10 level suggests a relationship between frozen shoulder and inflammation. Therefore, we think that the development of frozen shoulder is closely associated with both fibrosis and inflammation. The development of frozen shoulder involves a process of tissue stiffening caused by fibrosis, which is triggered by inflammation. Blocking the Smad4 gene has a positive effect on inflammation, the mechanism of which is of interest to us. To elucidate how Smad4 knockdown affects cellular fibrosis induced by TGF-β1 and joint fibrosis induced by chronic inflammation, we examined the activity of matrix metalloproteinases and the change in the Timp1 protein level, as well as the nuclear localization of Smad2/3. According to the in vitro experimental data, Timp1 increased and the activity of MMP decreased with TGF-β1 induction, and Smad4 gene silencing noticeably suppressed the increase in Timp1 protein and the decrease in MMP activity. We also measured Timp1 levels and MMP activity in an in vivo experiment and qualified phosphorylated Smad2/3 in cellular nuclei. In contrast to the control group, the model group exhibited an increase in the Timp1 level and a decrease in MMP activity, which were alleviated by Smad4 silencing. As is well known, Smad4, a member of the Smad family, forms complexes with phosphorylated Smad2/3 to facilitate their nuclear translation. Smad2/3 then binds to gene promoters to regulate downstream genes. Therefore, we inferred that Timp1 may be one of the target genes of Smad2/3 and that Smad4 silencing inhibits Timp1 expression and, in turn, increases MMP activity to suppress or retard tissue stiffening. To prove the hypothesis, we isolated the nuclear protein from the synovial tissue and assessed the change in phosphorylated Smad2/3 in the nuclei. Unlike in the control group, phosphorylated Smad2/3 accumulated in the nuclei in the model group, which was impaired by Smad4 knockdown. Therefore, Smad4 silencing interrupted the TGF-β/Smad pathway by inhibiting the nuclear translocation of Smad2/3 to suppress fibrosis mediated by the pathway, which may be the regulating mechanism for Smad4 silencing to inhibit frozen shoulder caused by fibrosis. Whether Smad2/3 regulates the transcription of Timp1 will be a part of our further study.

The present study showed that Smad4 silencing can inhibit fibrosis of synovial tissues and cells and produce a positive treatment effect in frozen shoulder, which is mainly characterized by fibrosis. All these results indicated that frozen shoulder causes an inflammatory response in the joint cavity and concomitant fibrosis. Smad4 silencing significantly inhibits the increase of these proteins, indicating that it suppresses joint inflammation and the fibrosis process of relevant tissues. Three weeks of medication also increased movement range of the joint and the volume of the joint capsule, and the difference was statistically significant compared with the model group. This shows that Smad4 silencing can postpone or reverse the joint stiffness caused by frozen shoulder. Smad4, therefore, may be a promising gene target for the treatment of frozen shoulder, although further study on the mechanisms is required. In the long run, we will assume that Smad4 is a target for gene therapy to treat frozen shoulder, which requires more work on the mechanisms, such as the BMP-2/Smad pathway, which is another regulating pathway in frozen shoulder. We will also upgrade the gene delivery approach to make it safer and more efficient.

## References

[1] Piersma B, Bank RA, Boersema M. Signaling in Fibrosis: TGF-β, WNT, and YAP/TAZ Converge. Front Med (Lausanne). 2015; 2:59. 26389119 10.3389/fmed.2015.00059 PMC4558529

[2] Li Y, Lua I, French SW, Asahina K. Role of TGF-β signaling in differentiation of mesothelial cells to vitamin A-poor hepatic stellate cells in liver fibrosis. Am J Physiol Gastrointest Liver Physiol. 2016;310(4):G262–72. 10.1152/ajpgi.00257.2015 26702136 PMC4754741

[3] Asghari MH, Hobbenaghi R, Nazarizadeh A, Mikaili P. Hydro-alcoholic extract of Raphanus sativus L. var niger attenuates bleomycin-induced pulmonary fibrosis via decreasing transforming growth factor β1 level. Res Pharm Sci. 2015;10(5):429–35. 26752991 PMC4691963

[4] Hongyun Li, Shiyi Chen, ZhaiWeitao and Chen Ji-wu. Expression of transforming growth factor-β and metalloproteinases in joint capsule of frozen shoulder. Journal of Shanghai Jiaotong University (Medical Science) Chinese. 2009;29 (11): 1363–1366.

[5] Hao J, Ju H, Zhao S, Junaid A, Scammell-La Fleur T, Dixon IM. Elevation of expression of Smads 2, 3, and 4, decorin and TGF-beta in the chronic phase of myocardial infarct scar healing. J Mol Cell Cardiol. 1999;31 (3): 667–678. 10198196 10.1006/jmcc.1998.0902

[6] Kopp J, Preis E, Said H, Hafemann B, Wickert L, Gressner AM, et al Abrogation of transforming growth factor-beta signaling by Smad7 inhibits collagen gel contraction of human dermal fibroblasts. J BiolChem. 2005;280 (22): 21570–21576. 10.1074/jbc.M502071200 15788410

[7] Goto Y, Manabe N, Uchio-Yamada K, Yamaguchi-Yamada M, Inoue N, Yamamoto Y, et al Augmented cytoplasmic Smad4 induces acceleration of TGF-beta1 signaling in renal tubulointerstitial cells of hereditary nephrotic ICGN mice with chronic renal fibrosis; possible role for myofibroblastic differentiation. Cell Tissue Res. 2004;315 (2): 209–221. 14615933 10.1007/s00441-003-0824-z

[8] Kanno A, Sano H, Itoi E. Development of a shoulder contracture model in rats. J Shoulder Elbow Surg.2010;19 (5): 700–708. 10.1016/j.jse.2010.02.004 20452246

[9] Hand GC, Athanasou NA, Matthews T, Carr AJ. The pathology of frozen shoulder. J Bone Joint Surg Br. 2007; 89 (7): 928–932. 17673588 10.1302/0301-620X.89B7.19097

[10] Omari A, Bunker TD. Open surgical release for frozen shoulder: surgical findings and results of the release. J Shoulder Elbow Surg.2001;10 (4): 353–357. 11517365 10.1067/mse.2001.115986

[11] Jiwu Chen, Shiyi Chen and Peng Zhang. Anatomic study of rotator internal and clinical significance. Chinese Journal of Clinical Anatomy. (Chinese) 2004;22 (1): 55–57.

[12] Schnaper HW, Hayashida T, Hubchak SC, Poncelet AC. TGF-beta signal transduction and mesangial cell fibrogenesis. Am J Physiol Renal Physiol.2003; 284 (2):F243–252. 12529270 10.1152/ajprenal.00300.2002

[13] Massagué J. TGFbeta in Cancer. Cell. 2008;134 (2): 215–230. 10.1016/j.cell.2008.07.001 18662538 PMC3512574

[14] Pennison M, Pasche B. Targeting transforming growth factor-B signaling. CurrOpinOncol. 2007;19 (6): 579–585. 10.1097/CCO.0b013e3282f0ad0e PMC2640227 17906455

[15] Hao J, Ju H, Zhao S, Junaid A, Scammell-La Fleur T, Dixon IM. Elevation of expression of Smads 2, 3, and 4, decorin and TGF-beta in the chronic phase of myocardial infarct scar healing. J Mol Cell Cardiol. 1999;31 (3): 667–678. 10198196 10.1006/jmcc.1998.0902

[16] Kopp J, Preis E, Said H, Hafemann B, Wickert L, Gressner AM, et al Abrogation of transforming growth factor-beta signaling by Smad7 inhibits collagen gel contraction of human dermal fibroblasts. J BiolChem. 2005;280 (22): 21570–21576. 10.1074/jbc.M502071200 15788410

[17] Goto Y, Manabe N, Uchio-Yamada K, Yamaguchi-Yamada M, Inoue N, Yamamoto Y, et al Augmented cytoplasmic Smad4 induces acceleration of TGF-beta1 signaling in renal tubulointerstitial cells of hereditary nephrotic ICGN mice with chronic renal fibrosis; possible role for myofibroblastic differentiation. Cell Tissue Res. 2004; 315 (2): 209–221. 14615933 10.1007/s00441-003-0824-z

[18] Foster W, Li Y, Usas A, Somogyi G, Huard J. Gamma interferon as an antifibrosis agent in skeletal muscle. J Orthop Res.2003; 21 (5): 798–804. 12919866 10.1016/S0736-0266(03)00059-7

